# A response to “Surveillance is not safety: a response to Dewa and colleagues’ paper about passive remote monitoring technology (Oxevision)”

**DOI:** 10.1186/s12888-026-08174-y

**Published:** 2026-05-28

**Authors:** Lindsay H. Dewa, Jonathan D. Hafferty, Robert Bates, Kevin Towers, Paul Aylin

**Affiliations:** 1 National Institute for Health Research Imperial Biomedical Research Centre, Imperial College London, London, UK; 2 School of Public Health, Imperial College London, Wood Lane, London, W12 0BZ UK; 3 West London NHS Trust, London, UK

**Keywords:** Oxehealth, Passive monitoring, Digital technology, Psychiatry

We welcome feedback from the authors Porter and Edwards through their comment on our paper: “*A service evaluation of passive remote technology for patients in a high-secure forensic psychiatric hospital: a qualitative study*” [1]. We aimed to gain a deeper understanding of the use of Oxehealth in one high-secure hospital from staff and patient perspectives. Our study was informed by staff and in-patients with lived experience of mental health difficulties, and of using Oxehealth (Oxevision) across the design, recruitment and data collection, analysis and interpretation project stages [2].

As the authors’ state, we report on patients’ experiences of paranoia and anxiety and the psychological impact of Oxehealth (Oxevision) across two themes “big brother syndrome” and privacy and dignity. Within these themes, we reported patient concerns about being watched in general, whether it was through Oxehealth or physical checks. For example, we report on some patients’ concerns about invasion of privacy, violation of their dignity, and that some patients feel embarrassed being watched by staff. We did not recommend a trial of Oxevision to replace in-person checks. We suggest the possibility of a feasibility and pilot study comparing Oxehealth (Oxevision) on patient safety with and without physical checks. This seems appropriate considering the concerns patients raised about physical checks on patients’ sleep and consequently their mood, wellbeing and quality of life.

The authors also mention that we misrepresent the functions of Oxevision when we state, “*most staff reported that they understood why patients felt they had no privacy*,* and they did their best to maintain it […] [by] trying to reassure the patients that the camera is not recording them all the time”.* Our reported findings are taken from the data we had, and we must report on what the staff and patients told us. Indeed, the authors go on to say that we highlight “a lack of knowledge and understanding about Oxevision amongst both staff and patients”, and how it operates. We also highlight in the discussion that there is therefore a need to provide better education about Oxehealth to improve patient and staff understanding, particularly with the camera operation.

The authors state we “did not seek” ethical approval for the study. This is incorrect. We did *seek* ethical approval, but we followed guidance through the Health Research Authority decision tool that deemed our study as not research. It was identified as a service evaluation because the Oxehealth technology was already operational within the psychiatric hospital. This was confirmed by the R&D department at Broadmoor Hospital within West London NHS Trust. As the authors state, we mention the “legal basis” of Oxehealth in the introduction. We have presented an overview of how Oxehealth is in line with, and details of the legislation and guidelines (UK Protection Act 2018, European Convention on Human Rights – Article 8), and an overview of camera protocols. However, it was not the aim of our study to explore the “legal issues” of Oxehealth (Oxevision) with patients and staff. The interview topic guide was co-produced with inpatients with Oxehealth technology experience. The potential legal concerns of Oxevision (Oxehealth) were not explicitly identified as an area of discussion within the interview topic guide. All participants gave informed consent.

To conclude, we report that Oxehealth technology was *mostly* considered acceptable by staff and patients, but that there are still concerns about its impact on privacy and that there is a delicate balance between this and patient safety. As the authors state, we mention how Oxehealth failed to detect the deterioration of a patient’s condition before their death in the findings and consider this finding within the discussed point that improvements are needed in Oxehealth (Oxevision) going forward, including improved accuracy in detecting deterioration. This is in line with the findings of the paper.

Thank you for considering our response to the comment.

## Data Availability

No datasets were generated or analysed during the current study.

## References

[1] Dewa LH, Broyd J, Hira R, Dudley A, Hafferty JD, Bates R, Aylin P. A service evaluation of passive remote monitoring technology for patients in a high-secure forensic psychiatric hospital: a qualitative study. BMC Psychiatry. 2023;23(1):946. 38098066 10.1186/s12888-023-05437-w PMC10722773

[2] Ting L. Diagram outlining the research process and how patient and public involvement can be embedded in it. In.: NIHR ARC West; 2023.

